# Observations of evidence-based medicine in general practice

**DOI:** 10.1007/s40037-013-0078-8

**Published:** 2013-09-04

**Authors:** Sandra E. Zwolsman, Nynke van Dijk, Margreet Wieringa de Waard

**Affiliations:** Department of General Practice/Family Medicine Academic Medical Center, University of Amsterdam, Meibergdreef 9, 1105 AZ Amsterdam, the Netherlands

**Keywords:** Evidence-based medicine, General practice, Postgraduate education, Qualitative research

## Abstract

No objective measures are available for assessing the practice of evidence-based medicine (EBM) in general practitioner (GP) trainees, as there is no description of the EBM behaviour that is expected from trainees. As a first step to do so, we aimed to identify which expressions of EBM (defined as the integration of evidence, clinical experience and patient situation) can be observed in daily GP practice. Secondly, we aimed to identify which considerations GPs had regarding EBM but did not share with the patient during consultations. We performed a qualitative study, in which GPs were observed during and interviewed after clinical consultations, with a focus on expressions and considerations related to EBM during clinical decision-making. We observed 147 consultations by 34 GPs (17 trainers and 17 trainees). EBM behaviour was rarely visible in GPs’ decision-making. When interviewing the GPs, we found that aspects of EBM that played a role in decision-making were not discussed with the patient. Explicit consideration of all aspects of EBM would make EBM measurable and GPs more aware of the foundations of their decisions. EBM behaviour is difficult to observe during GP consultations and therefore cannot be assessed through observations alone.

## Introduction

When aiming for patient-centred medicine and true shared decision-making, as is currently advocated in the medical literature [1] and is in line with EBM, a fundamental step in this process is to share the relevant information between doctor and patient and to reach consensus on a decision [2]. This implies that we expect physicians to actively share evidence, experience and preferences with the patient and to discuss the patient’s situation and preferences.

Commonly, training and assessment in EBM focuses solely on the knowledge and skills that are required to retrieve and use new evidence. Also, in a recent systematic review, we showed that no objective measures are available for assessing the actual practice of EBM by GPs [3]. Application of EBM is studied only by measuring the frequency of application of evidence. This results in an incomplete understanding of the full concept of EBM, which is about the integration of evidence, the physician’s expertise, and the patient’s situation and preferences [4, 5]. Competence-based assessments [6] are needed to stimulate adequate EBM behaviour. But it is not known on which behaviour these assessments should focus, as the ‘EBM behaviour’ that can be expected from trainees in daily clinical practice has not been described. A first step in the development of an observation instrument to assess EBM behaviour in daily clinical practice is to describe the EBM behaviour observed in daily clinical practice.

### Theoretical framework

EBM has been defined in varying ways [7]. Sackett et al. [8] defined it as ‘the conscientious, explicit, and judicious use of current best evidence in making decisions about the care of individual patients’. Several additions have been made to this definition on factors related to the physician and the patient [9–11].

We studied the literature on the concept and definition of EBM to formulate a comprehensive definition of EBM. The definition by Dawes et al. [9] was selected for this study. They defined EBM as ‘the conscientious, explicit and judicious use of current best evidence, in combination with the physician’s clinical expertise and the preferences and situation of the patient in making decisions about the care of individual patients’ [9].

Furthermore, Charles et al. [7] stated that EBM can be seen either from a theoretical approach—in which the five EBM steps (ask, access/acquire, appraise, apply, audit) [9] play a leading role—or from a practice-oriented approach, in which the combination of the three aspects of EBM is of relevance [7]. In this study, we focused on this practice-oriented approach. EBM behaviour then requires an integration in practice of the evidence itself, the physician’s preferences (based on experience and personal expertise) [12], and the patient’s preferences (i.e. regarding treatment options) and situation [8, 11, 13]. We therefore consider EBM competence in practice to entail the clinical decision-making during patient encounters in which all three of the above-mentioned aspects are taken into account.

Evidence is the current best available information from clinical care research [7]. For GPs, the best evidence will usually be in the guidelines. However, when the guidelines do not apply, the current best evidence should be derived, preferably using the five EBM steps [8, 9, 11]. Knowledge of the current best evidence is a prerequisite for the practice of EBM. Physician preferences are the ‘proficiency and judgement that has been acquired through experience and practice’ [7]. These are a combination of the physician’s basic skills and personal experience [11]. Patient preferences and situation are ‘perspectives, beliefs, expectations and goals for health and life’ and his or her clinical state and circumstances [7, 11].

As a pilot study, four observers (one GP and three epidemiologists, all EBM experts) watched a random sample of videoed patient consultations (*n* = 47) of first-year GP trainees. The observers discussed which EBM behaviour was visible in the video consultations and whether the three aspects of EBM could be identified. Based on these observations, we expected trainees to behave differently than experienced GPs due to less expertise and recent EBM training, and felt we should observe consultations of both groups to obtain a broad view of the practice of EBM. Furthermore, it was suggested that questioning the observed GPs would add the option of testing whether the observations were correct and whether the GP had any considerations which were not shared with the patient.

We therefore observed GPs making decisions in clinical practice aiming to create an overview of expressions regarding the three aspects of EBM (evidence, physician preferences, and patient preferences and situation). The second aim was to explore which considerations GPs had regarding clinical decisions, related to these three aspects, which were not shared with the patients.

## Methods

In this qualitative study, experienced GPs and GP trainees were observed during clinical consultations to study their expressions related to evidence-based decision-making. The study was conducted among GP trainers and GP trainees allied to the General Practice speciality training programme of the Academic Medical Center, University of Amsterdam.

### Context

In the Netherlands, GPs serve a specific role as gatekeepers for patients of all age groups. All Dutch citizens are registered to a GP, and they need a GP referral to see a medical specialist. For common clinical symptoms, GPs are expected to work according to the almost 100 GP guidelines, issued and updated by the Dutch College of General Practitioners. GPs are reregistered every 5 years based on clinical experience and a minimum amount of additional training. This training has no pre-specified content; EBM training is therefore not obligatory.

The GP speciality training is a 3-year programme comprising both formal training and training in practice. In their first and third years, GP trainees work in practice under the supervision of a GP trainer. In their second year, trainees participate in 3- to 6-month clinical traineeships. One day a week, trainees receive formal education on all CanMeds competencies [20] at the training institute. The EBM training consists of training in searching and appraising clinical evidence, performing a critically appraised topic (CAT) assignment, and journal clubs in which the focus is on integrating evidence in daily clinical practice.

### Data collection

Relevant aspects of EBM derived from the theoretical framework of EBM and the video observations were used to create an observation form (Appendix) containing the following items: patient characteristics, the policy of the physician, and observed expressions of EBM, specified for evidence, the physician and the patient (text boxes for quotations).

The observers annotated all information relating to the study aims. Only consultations in which patients presented new problems were included, as follow-up consultations regarding earlier medical decisions were considered less relevant for the observations of EBM behaviour during clinical decision-making in daily practice. All expressions about each aspect of EBM were written down. Two observers carried out the observations in practice. Both observers hold an MSc in evidence-based practice, and perceive the concept of EBM as given above. Before starting the observations, the observers reached consensus about the theoretical framework, the aims of the observations and their role during the observations. The role of the observer during the consultations was a non-participating and motive-hidden role: the observer did not interfere with the interaction between the GP and the patient, and in order to preclude socially acceptable behaviour, did not give information about the purpose of the observation [14].

We selected the study sample purposively, ensuring that we included a broad range of characteristics of GP practices, namely the location of the practice as related to the patient population, the number of GPs in the practice and the gender of the physicians, as we expected that the characteristics of both the patient population and the physicians would influence the manner in which EBM was used in clinical practice. We observed first- and third-year GP trainees and GP trainers, as we expected that the amount of experience and training influences expression of EBM use [15].

We interviewed all GPs directly after the observation session. The interview took place after a maximum of 6–7 consultations (1 h of clinical consultations, based on the availability in the GP’s agenda) to avoid recollection bias. The interviews lasted for approximately 15 min, and started with an introduction on the purpose of the study and the definition of EBM used for the study. Aspects of EBM that had been observed during the consultations were discussed with the GPs to verify the validity of the observations and to clarify the meaning of the observed expressions [16]. For instance, when one GP said ‘We know this’, the observer asked: ‘Where do you know this from?’ Then GPs were asked which EBM-related considerations they had made that the observer would not have perceived. For instance, the observer asked a GP who had not asked the patient about his preferences to what extent the preferences and situation of the patient had played a role in the decision-making. The comments of the GPs were annotated next to the field notes taken during the observations.

### Ethical considerations

All the GPs gave their written informed consent and were free to refrain from participating in the study. The head of the GP speciality training programme gave permission to carry out this study. The ethics review board of the Netherlands Association for Medical Education approved of the study (NVMO–NERB number 114).

### Analysis of the data

Observations in clinical practice were continued until data saturation. In between sessions, the observations and interviews were discussed by the observers and other team members at regular intervals to assess whether new observations were still being made and the definition of EBM was used similarly by the observers. Observation forms and notes from the interviews were analyzed by reading the field notes and the use of open coding following five steps: [17] sorting out annotations of the observations and preparing them for analysis; creating a rough overview of the identified categories of observations; coding; performing a detailed analysis; and analyzing outcomes. The definition of EBM as described in the introduction was considered as leading during the analysis. The analyses were performed by two researchers: a university teacher (MD, PhD, educationalist) in EBM in the GP speciality training programme, and a PhD student involved in this project. Both researchers are EBM experts through research experience and education, and define EBM as stated in this article. Differences in coding were resolved by discussion, when necessary in the presence of another team member, until consensus was reached.

Codes in brackets ([]) refer to the observed GP and number/letter of consultation.

## Results

Between February and June 2012, we observed 147 clinical consultations by 34 GPs (17 trainers and 17 trainees). The median number of relevant observations was 4 (range 1–7) before the interview took place. Of the trainers 59 % were male, of the trainees this was 29 %. The experience of trainers varied from several up to 20 years of training experience. Practice size varied from one to five GPs.

The consultations concerned patients from all age groups and with different symptoms. Of the patients, 57 % were female, and 22 % were children (<16 years, 10 % adolescents (16–25 years), 50 % adults (25–65 years) and 18 % elderly patients (>65 years). Symptoms covered all the body systems. Common complaints (presented in >10 consultations) were ear, nose and throat infections, skin problems, abdominal symptoms and arm or leg problems. Some patients presented with more than one complaint.

### General findings

A first finding when analyzing the field notes taken during the observations was that the consideration and integration of all three components of EBM was observed during only one consultation [25A]. In a third of the consultations none of the three aspects of EBM were observed. In these cases no arguments regarding the decision-making were given by the GP; information and decisions were stated as facts without explicitly mentioning the underlying considerations on any of the three EBM aspects. When a GP expressed knowledge as a basis for the decision, no differentiation could be made by observation as to whether evidence or experience was used. In another third of the consultations only patient preferences were more or less explored. In over 80 % of these cases this was limited to either one open question in terms of: ‘what can I do for you?’, or the closed question ‘do you agree with my suggestion’. In less than 20 % of these cases the personal situation of the patient was explicitly taken into account during the clinical decision-making: ‘because you are an active sportswoman I will refer you earlier’ [24E]. If the patient was asked for an opinion this was mainly restricted to detailed practical choices, such as which hospital to refer the patient to [12D]. In the other consultations, expressions regarding either evidence <10 %, clinical experience <2 % or two aspects of EBM were observed.

We observed that the use of the different aspects of EBM was mainly related to the specific decision-making style of the GP, and not to the kind of patient or health problem. For instance, GPs either actively sought evidence during almost every consultation, or never did so. Distinctive differences were observed between the expressions of EBM between experienced GPs and GP trainees. During the interviews, experienced GPs referred to the fact that they had known their patients for years, and therefore know their preferences, and to their long-lasting clinical experience, where trainees obviously did not. Trainees consulted their trainer and medical specialists where experienced GPs were not observed to do so. GP trainees were more inclined to search for evidence than experienced GPs were.

There was no clear pattern in expressions of EBM related to gender. There was, however, clear coherence in how the GPs dealt with high-risk cases (patients in whom chronic or malignant illnesses were suspected) or medical urgencies. In such cases, GPs did not generally share their suspicions with the patient until more information became available [22D, 24C, 27A]. For instance, one GP stated that she did not want to tell the patient the outcomes of her own physical examination until further tests had been done regarding the patient’s health [24D].

The interviews revealed that GPs do not communicate all considerations to their patients. Below we describe the specific observations and interview results regarding the three aspects of EBM.

### Patient preferences and situation

The patient’s situation was observed to be mainly considered during the decision-making when it was related to minor details, for instance by discussing how much health insurance companies pay towards the costs of an intervention [12C], about methods of administration (fluid/gel) [22A] or which hospital the patient is referred to [12C]. GPs explicitly included the situation of the patient by saying ‘I know you don’t tolerate that medicine’ [7.5] or ‘As it worked in the past …’ [24A]. However, the patient’s situation was not always included [11C]; For instance, a GP advised a plasterer with knee problems to take it easy [5.2], without mentioning the influence of that advice on his employment. Discussions of the pros and cons of treatment options were observed, but rarely.

If GPs did not agree with the patient’s treatment preferences, they said so: ‘Although I’m not very keen on giving you the medicine, it’s your choice and I’m prepared to prescribe it to you’ [25A]. The GP sometimes included the patient in decision-making in order to assure the patient, by making such statements as ‘If doing nothing makes you nervous, I could prescribe you medication instead’ [17] or—without a clear preceding clinical question—‘I want to assure you that nothing is wrong, so I’ll do this test’ [5.5].

GPs also regularly verified whether the patient agreed with the proposed treatment, although this did not necessarily mean that the patient’s preferences were taken into account [20B].

When a GP was uncertain regarding the diagnosis or treatment of a patient, the patient was explicitly included in the decision-making process, seemingly to share responsibility. In those situations, the patient’s preferences had a prominent influence on the decision-making: ‘What do you want?’ [22D]; ‘What shall we do? You tell me.’ [25C] When a patient had a clear clinical question, including suggestions or a preference for treatment, the GP was generally inclined to follow the patient’s suggestions [21D].

The interviews added to the observations that also negative opinions on patients or the patient’s situation play a role in the decision-making process, which were not shared with the patient. Factors such as frequency of attendance [12C] or presumed character of the patient (‘She’s a nag’ [17]) were mentioned as reasons for clinical decisions. Also, GPs base decisions on unverified interpretations and ideas about the situation and wishes of the patient. For instance, before the start of one consultation, the GP said: ‘This patient will get an antibiotic prescription’ [12E]. If a patient was really ill or experiencing difficulties in daily life [22D]—whether or not verified by the patient—this was given as a reason to give immediate treatment even when not indicated in the guidelines.

### Evidence

Before consultations, some GP trainees searched online (Google), or consulted the guidelines to find information regarding the patient’s clinical problem, when this was noted by the receptionist.

During patient consultations, we observed an active search for evidence during less than 3 % of the consultations. If evidence was sought, it was easily retrievable evidence, such as required dosages of medication [2.4]. Only one clear expression of EBM to a patient on available evidence was observed: ‘There are guidelines, which say…’ [28B]. On one occasion, a GP told a patient that she needed to search for more information (‘I have to look at my books’) [26B]. Absence of evidence was sometimes used to convince a patient; for instance, one patient wanted anticoagulants whereas they were not indicated (‘There is no evidence that you will live longer’) [17].

During the interviews GPs stated that they did not always know the source of the evidence they used. For example, GPs told the interviewer: ‘I think research has been done’, ‘It might be in the guidelines’, ‘Sometimes I am guessing’ [1.2] and ‘I do not know the source of this’ [12B]. In one case, the GP told the interviewer: ‘There’s no evidence about this’ [3D]. GPs sometimes consciously deviated from the evidence, for reasons related to the situation of the patient (‘We usually give this medicine, but in your case …’) [16, 26A.] During the interviews, several GPs felt the need to look up the evidence [16C, 28A, 29A], or even revise the clinical decision.

### GP preferences and experience

GPs explicitly shared their own preferences or experience with patients in less than 2 % of the observed consultations. During one observation, a GP clearly expressed his personal preference by telling his patient: ‘My preference is this. What do you think about that?’ [16] GPs did make statements such as ‘I’m 100 % certain that you don’t have rheumatoid arthritis’ [7.5]. This kind of expression seemed to be derived from personal experience, although this was not explicitly mentioned as such.

When the clinical problem of a patient was unclear, GPs said that they had inadequate experience to make a clinical decision (‘I don’t know. I’ll refer you to a specialist.’). This was confirmed during the interviews, although it was often not mentioned to the patient: ‘I don’t have the illusion of knowing it better than a specialist’ [12D].

Practical experience and knowledge obtained during speciality training [6.3;7.3] were mentioned during the interviews as the basis for clinical decisions. Clinical experience such as ‘A viral infection lasts for 5 days’ [10C] was presented as general knowledge. Although not explicitly mentioned to the patient, the experiences that GPs have had with other patients with similar health problems play a role in decision-making: ‘It’s not evidence based, but it does work better’ [14E]. A positive experience in other patients was expressed to patients: ‘This worked for another patient …’ [2C].

## Discussion

In this study we aimed at identifying which expressions of the three components of evidence-based decision-making (evidence, physician’s preferences, and patient’s preferences and situation) are demonstrated in clinical practice, and at determining which EBM considerations are not shared with patients but do play a role in decision-making in clinical practice.

Our main finding is that EBM behaviour is limited in daily clinical practice, since expressions regarding the use of aspects of EBM in the decision-making of GPs are scarce, especially when related to clinical experience or evidence. Although our study shows that at times one or more of the three aspects of EBM are expressed as the basis for decision-making, most GPs do not explicitly use expressions related to the three aspects of EBM during patient consultations. When interviewing the GPs, we found that aspects that we did not observe during the consultations played a role in decision-making, but GPs do not share the source on which they base their decisions with their patients. This is in contrast with the content of formal education and the concept of shared decision-making [2, 3].

This finding is important for the development and possible use of instruments to assess EBM behaviour or competence in clinical practice. As we now know that expression of EBM behaviour is rare in both experienced GPs and GP trainees, the first discussion should be about the behaviour that we expect from physicians, and the possible consequences of this expected behaviour for the patient, the physician and the educational system.

According to the principles of shared decision-making, we expect physicians to actively share considerations regarding evidence, experience and the patient’s preferences with the patient. However, this approach is mainly applicable in situations in which there is a debatable trade-off between benefit and harm, or when significant effort on the part of the patient is required for a successful result [3].

One could question whether this approach is feasible during the short time available for GP consultations. Fortunately, most GPs know their patients and their situation very well. In this, the advantage for the GP is that interventions can more easily be adjusted to the patient, without discussing all the considerations at every moment. This could, however, also be a pitfall, because in this study we found that the patient’s preferences and situation were not always checked. Elwyn and colleagues confirm that GPs generally do what they consider to be best for the patient without explicitly including the patient’s preferences [18].

In addition, research into whether patients actually want to be included in decision-making is conflicting: some studies conclude that patients want to be included [19], whereas others conclude the opposite [20, 21]. However, all these studies agree that patients want to have more information about their illness and treatment [20, 21]. Barry and colleagues stated that not fully informing the patient can result in treating patients who would not have wanted treatment had they been fully informed [19].

We found a specific situation in which patient preferences were not considered, namely when the patient required immediate medical care, or the gut-feeling of the GP indicated a high risk of serious disease [22]. Barry and colleagues also stated that patient preferences are of limited value when ‘one superior path’ has to be taken to treat the patient [19]. In that case, only the patient’s situation is to be integrated into the decision-making.

GPs frequently did not know the source of the evidence. This finding is confirmed by Gabbay al. [23], who found that tacit knowledge is commonly used. Subsequently, it could be unclear to GPs whether their knowledge is derived from experience or from previously acquired evidence; [23] if that is the case, they could be uncertain about whether their knowledge is up to date. Sharing their considerations regarding evidence, experience and the patient’s situation with their patients could enhance physicians reflections on the knowledge used and its critical application and make GPs more aware of the foundation of their decisions.

Since EBM behaviour is currently rarely visible in practice, it seems hard to assess EBM competence by practice observations alone [18]. GP trainees could be stimulated by means of training and assessment to express their considerations regarding decision-making during patient consultations whenever such is possible. To do so, they should be trained in shared decision-making and stimulated to reflect on their decisions. A first step in adapting education would be to teach GPs what EBM behaviour entails—in conformity with the definition given in this study—and what competences are needed to accurately apply EBM in clinical practice. Otherwise, observation of EBM behaviour could be performed with, for instance, simulated patients following structured scripts about topics relevant to all three EBM/shared-decision-making [2, 3] skills. This could stimulate GPs to use all the aspects of EBM, to reflect on how they practise EBM, and to find an optimum balance between explicit and implicit use of EBM.

### Strengths and limitations

This study was the first to observe the aspects of EBM in clinical decision-making during patient encounters. There were some limitations to the execution of this study. First, although the study sample comprised a diverse selection of GPs and we obtained information until data saturation occurred, all GPs were allied to an academic institution. GP trainers and trainees receive supplementary education and may have a special interest in EBM. However, as the purpose of this study was to explore the possible expressions of EBM behaviour and as data saturation was reached, we do not believe that the inclusion of this population led to the observation of different, but maybe more, expressions of EBM.

This study was conducted in the Netherlands. We found that evidence was seldom explicitly explained to patients, and this—or taking the preferences of the patients into account—may be different in other countries. Our findings may have been influenced by culture: the health care system, or the habits of GPs or observations made in GP practices in other countries may reveal different expressions of EBM.

Social desirability bias might have occurred during the interviews after the purpose of the study had been revealed. Their answers could have been influenced by the feeling that the observers expected behaviour, while in fact the considerations given played no role in the decision-making process. This was partly prevented by first performing the observations without explaining the study aim to the participants. On the other hand, behaviour shown after the purpose of the study had been revealed and discussed might have resulted in additional behaviours not common, though possible, in daily practice.

## Essentials

- Expressions of EBM behaviour are limited in daily GP practice.
- The explicit combination of evidence, the doctor’s experience and the situation and preferences of the patient is rarely observed.
- GPs often do not share the source of their decisions with their patients.
- Possibilities of EBM assessment in clinical practice are therefore currently limited.
- A discussion on the expected EBM behaviour in trainees is needed.
